# MIF inhibits the formation and toxicity of misfolded SOD1 amyloid aggregates: implications for familial ALS

**DOI:** 10.1038/s41419-017-0130-4

**Published:** 2018-01-25

**Authors:** Neta Shvil, Victor Banerjee, Guy Zoltsman, Tom Shani, Joy Kahn, Salah Abu-Hamad, Niv Papo, Stanislav Engel, Jurgen Bernhagen, Adrian Israelson

**Affiliations:** 1 Department of Physiology and Cell Biology, Faculty of Health Sciences, Ben-Gurion University of the Negev, P.O.B. 653, Beer Sheva, 84105 Israel; 2 Department of Biotechnology Engineering, Faculty of Engineering, Ben-Gurion University of the Negev, P.O.B. 653, Beer Sheva, 84105 Israel; 3 The National Institute for Biotechnology in the Negev, P.O.B. 653, Beer Sheva, 84105 Israel; 4 Department of Clinical Biochemistry and Pharmacology, Faculty of Health Sciences, Ben-Gurion University of the Negev, P.O.B. 653, Beer Sheva, 84105 Israel; 5 Institute for Stroke and Dementia Research (ISD), Ludwig-Maximilians University, D-81377 Munich, Germany; 6 The Zlotowski Center for Neuroscience, Ben-Gurion University of the Negev, P.O.B. 653, Beer Sheva, 84105 Israel

## Abstract

Mutations in superoxide dismutase (SOD1) cause amyotrophic lateral sclerosis (ALS), a fatal neurodegenerative disease caused by the progressive loss of motor neurons in the brain and spinal cord. It has been suggested that toxicity of mutant SOD1 results from its misfolding, however, it is yet unclear why misfolded SOD1 accumulates specifically within motor neurons. We recently demonstrated that macrophage migration inhibitory factor (MIF)—a multifunctional protein with cytokine/chemokine activity and cytosolic chaperone-like properties—inhibits the accumulation of misfolded SOD1. Here, we show that MIF inhibits mutant SOD1 nuclear clearance when overexpressed in motor neuron-like NSC-34 cells. In addition, MIF alters the typical SOD1 amyloid aggregation pathway in vitro, and, instead, promotes the formation of disordered aggregates, as measured by Thioflavin T (ThT) assay and transmission electron microscopy (TEM) imaging. Moreover, we report that MIF reduces the toxicity of misfolded SOD1 by directly interacting with it, and that the chaperone function and protective effect of MIF in neuronal cultures do not require its intrinsic catalytic activities. Importantly, we report that the locked-trimeric MIF^N110C^ mutant, which exhibits strongly impaired CD74-mediated cytokine functions, has strong chaperone activity, dissociating, for the first time, these two cellular functions. Altogether, our study implicates MIF as a potential therapeutic candidate in the treatment of ALS.

## Introduction

Amyotrophic lateral sclerosis (ALS) is a progressive and fatal neurodegenerative disease, which belongs to the family of protein misfolding and protein aggregation diseases, and is characterized by the selective loss of upper and lower motor neurons. Approximately 10% of all ALS cases are familial, wherein most genes are inherited in an autosomal dominant manner^1^. About 20% of familial ALS cases are attributed to mutations in the gene encoding the cytoplasmic Cu/Zn superoxide dismutase (SOD1)^2^, and more than 150 mutations in SOD1 have been identified and described as causing ALS in a dominant manner^3,4^. Several hypotheses have been proposed to explain mutant SOD1-mediated toxicity, but the mechanisms responsible for motor neuron degeneration in ALS have not been fully elucidated. Phenotypically, mutant SOD1 toxicity in ALS is universally attributed to SOD1 misfolding and aggregation^5^, with a clear inverse correlation between mutant SOD1 misfolding and aggregation and disease duration in SOD1 patients^6–8^, suggesting that a gain of novel noxious function upon misfolding may be involved in the pathogenesis of ALS, as proposed previously^9^. In line with this suggestion, is the fact that insoluble aggregates in both familial and sporadic ALS cases are SOD1-immunoreactive^10,11^.

Unlike wild-type SOD1 (SOD1^WT^), mutant SOD1 has been shown to associate with mitochondria^12–17^ and/or the endoplasmic reticulum (ER)^18–21^ solely in tissues from the nervous system. Specifically, the association of mutant SOD1 with the ER has been implicated in ER stress induction^18–21^, and misfolded SOD1 association with mitochondria has been shown to directly bind the voltage-dependent anion channel-1 (VDAC1) and to inhibit its conductance of adenine nucleotides across the outer mitochondrial membrane^13^. This inhibition was found to be specific to spinal cord mitochondria and has not been observed in mitochondria extracted from unaffected tissues^13,22^. In addition, mutant SOD1 was shown to interact with, and alter the function of other components of the mitochondrial outer membrane, including Bcl-2^23^ and the protein import machinery^22^. Despite their potential to shed light on mutant SOD1-induced toxicity in ALS, the molecular factors that determine the cell specificity of misfolded SOD1 proteins, and which drive them to bind and accumulate solely within intracellular membranes of the nervous system, have not yet been identified.

Recently, macrophage migration inhibitory factor (MIF)—a multifunctional protein with cytokine/chemokine activity and cytosolic chaperone-like properties—was shown to inhibit mutant SOD1 misfolding on mitochondria and ER in vitro^24^ and in vivo^25^. At the same time, MIF levels were found to be extremely low within motor neurons^24,25^. In the current study, we used recombinant MIF and SOD1 mutants in a variety of in vitro and cell-based assays to determine the mode of action of MIF as a chaperone for misfolded SOD1. We report that MIF mutants that lack the intrinsic, evolutionarily conserved, tautomerase or oxidoreductase enzymatic activity are able to function as chaperones for misfolded SOD1 and to inhibit mutant SOD1-dependent neuronal cell death. Moreover, we show that the protective effects of MIF are associated with its trimeric conformation, and that MIF directly interacts with SOD1 and suppresses the formation of misfolded SOD1 amyloid aggregates by promoting the conversion of misfolded SOD1 into amorphous disordered aggregates.

## Results

### MIF inhibits mutant SOD1 nuclear export in NSC-34 cells

**Fig. 1:**
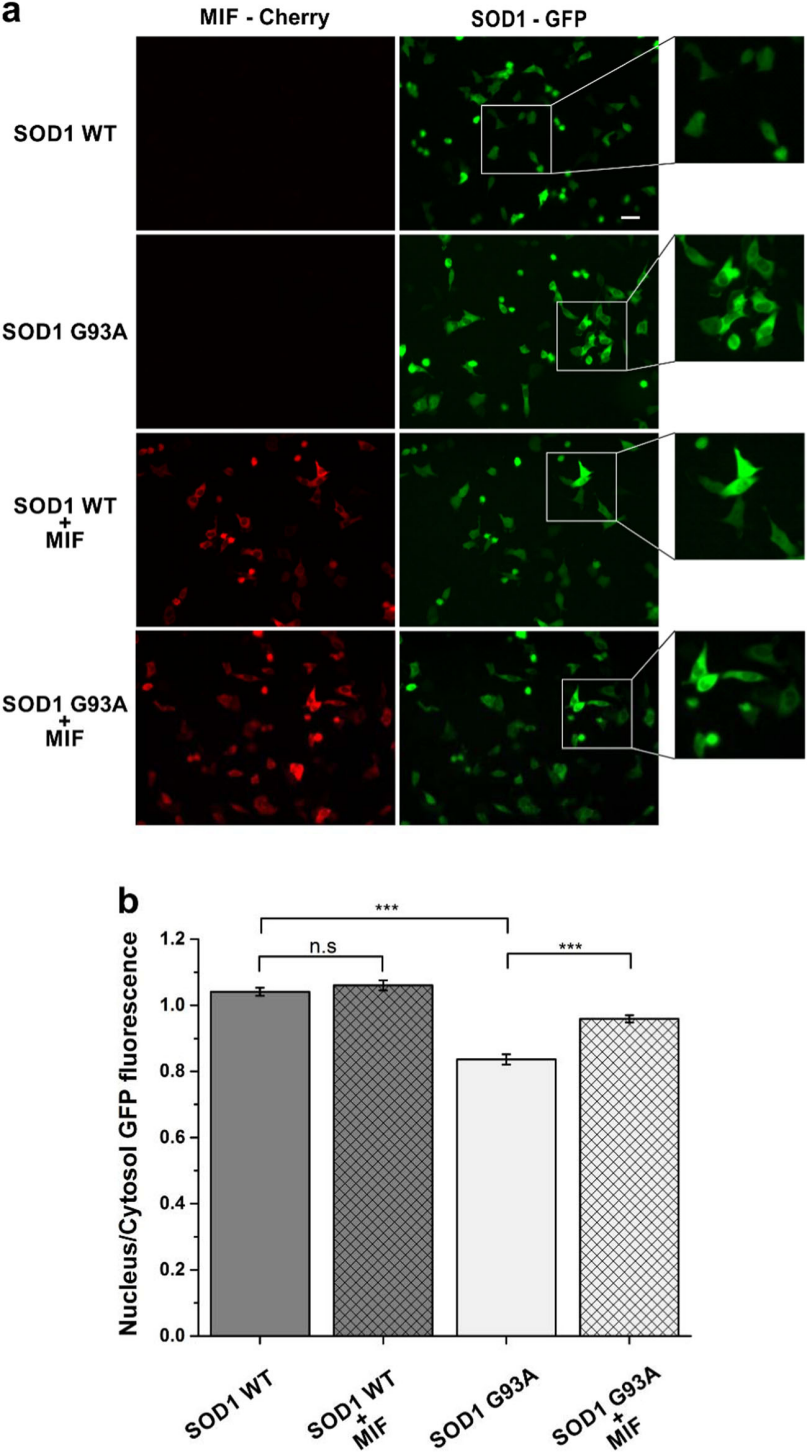
MIF inhibits misfolded SOD1 nuclear export in NSC-34 cells **a** NSC-34 cells were transfected with SOD1^WT^–EGFP or SOD1^G93A^–EGFP plasmid with or without co-transfection of MIF-Cherry. 24 h after transfection, between 57–60 images were recorded for each group. Scale bar, 25 µm. **b** GFP fluorescence levels in the nucleus and in the cytoplasm were quantified separately by NIS elements (NIKON) program and the ratio for each cell were taken for analysis. One-way ANOVA test was applied on the average of the different groups, all normal distributed, followed by Tukey post hoc test. ***p < 0.001

As previously shown, SOD1 is normally localized in both the cytoplasm and the nucleus of cells^26,27^. Recently, Zhong and colleagues showed that the misfolding of SOD1 exposes a nuclear export signal (NES)-like sequence, which is normally buried in correctly folded SOD1^WT^^27^, allowing the clearance of misfolded SOD1 from the nucleus by the nuclear export carrier protein CRM1. In accordance with these findings, we found that when SOD1^WT^–EGFP and mutant SOD1^G93A^–EGFP are expressed in motor neuron-like NSC-34 cells, SOD1^WT^ is evenly distributed between the cytoplasm and the nucleus while mutant SOD1^G93A^ shows predominantly cytoplasmic distribution (Fig. 1a, b). Expression of MIF in cells expressing SOD1^WT^ had no effect on the distribution of the SOD1^WT^–EGFP protein. However, expression of MIF together with the mutant SOD1^G93A^–EGFP, inhibited the nuclear clearance of misfolded SOD1 resulting in a more wild-type-like distribution of the mutant SOD1 protein (Fig. 1a, b).

### Recombinant MIF specifically suppresses the formation of mutant SOD1 amyloid fibrils and induces the formation of disordered amorphous aggregates

**Fig. 2:**
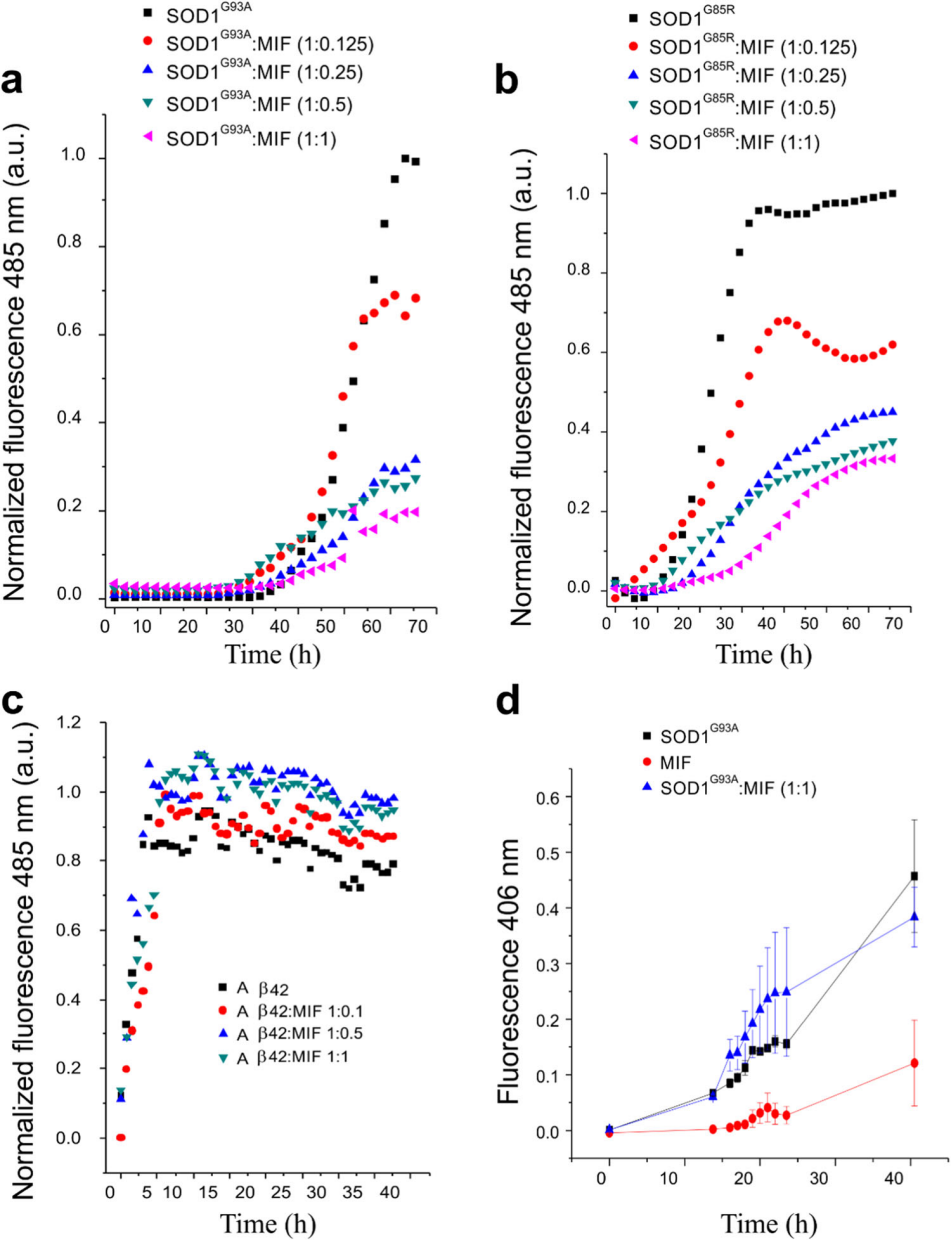
Recombinant MIF specifically suppresses mutant SOD1 amyloid fibril formation in a dose-dependent manner ThT fluorescence was monitored during the co-incubation of SOD1^G93A^ (50 µM) (**a**) or SOD1^G85R^ (50 µM) (**b**) with recombinant MIF at different molar ratios at 37 °C with continuous shaking. The values were normalized to the maximal ThT intensity elicited by SOD1^G93A^ or SOD1^G85R^ alone. **c** ThT fluorescence of the β-amyloid peptide (Ab42) (1.0 µM), incubated alone or with increasing concentrations of recombinant MIF, under the same conditions as in (**a**) and (**b**). Data points in **a**–**c** represent the average results from one representative experiment (performed in triplicates) of three independent experiments. **d** An increase in the turbidity measured at 406 nm indicates the formation of SOD1^G93A^ aggregates in solution during shake-incubation at 37 °C, in the absence (black) or presence (blue) of 10 μM MIF. The turbidity of MIF alone (10 μM; red) is also shown as a control. Of note, in this MIF concentration range (blue symbols) significant inhibition of ThT fluorescence elicited by SOD1^G93A^ or SOD1^G85R^ is seen.

We have previously shown that MIF can inhibit the accumulation of misfolded SOD1 in vitro and in vivo^24,25^. To test whether recombinant MIF can prevent the amyloid fibril formation of mutant SOD1, we expressed and purified MIF as described previously^28^ and measured its overall structural integrity and activity by using the hydroxyl-phenolpyruvate (HPP) tautomerase activity assay (Fig. S1). Incubating SOD1^G93A^ or SOD1^G85R^, another well-established misfolded SOD1 mutant, in the absence of recombinant MIF resulted in an exponential increase in thioflavin T (ThT) fluorescence (which correlates with amyloid aggregate formation) after a lag time of 30 or 15 h, respectively (Fig. 2a, b). In contrast, incubating mutant SOD1^G93A^ (Fig. 2a) or SOD1^G85R^ (Fig. 2b) in the presence of recombinant MIF strongly suppressed this increase in a dose-dependent manner. This effect was specific to amyloid aggregates of SOD1, as it was not observed when incubating the amyloid beta peptide Aβ_1-42_ with increasing MIF concentrations (Fig. 2c).

**Fig. 3:**
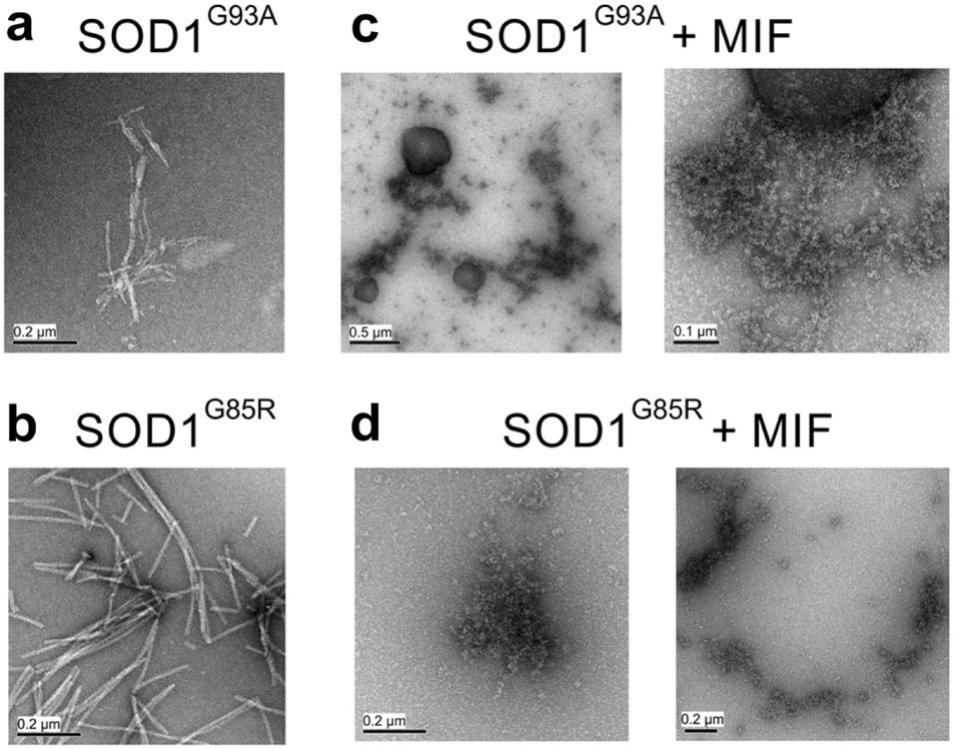
Recombinant MIF alters the morphology of SOD1^G93A^ and SOD1^G85R^ aggregates TEM images of a SOD1^G93A^ (50 μM) or SOD1^G85R^ (50 μM) solution after a 64-h incubation at 37 °C with continuous shaking. SOD1^G93A^ and SOD1^G85R^ were incubated either alone (**a**, **b**, respectively) or in the presence of recombinant MIF at a molar ratio of 1:1 (**c**, **d**, respectively).

To determine whether the presence of MIF reduces the total amount of SOD1 aggregates, we conducted a turbidity assay, in which all different forms of aggregates can be measured. Importantly, incubating the mutant SOD1 with MIF did not reduce the total amount of SOD1 aggregation (Fig. 2d) whatsoever, suggesting that MIF can induce misfolded SOD1 to aggregate into a species other than amyloid aggregates. Supporting this notion, transmission electron microscopy (TEM) imaging performed after incubating the SOD1 mutants with or without MIF revealed that, whereas mutant SOD1^G93A^ and SOD1^G85R^ form fibrous aggregates when incubated alone (Fig. 3a, b), incubating them with recombinant MIF switches the aggregation pattern to an amorphous disordered one (Fig. 3c, d). Incubating MIF alone produced very small aggregates, which were undetectable in the turbidity assay (Fig. 2d) or ThT analysis (Fig. S2).

### The conserved catalytic activities of MIF and its oligomeric transition are not required for suppressing the misfolding and toxicity of mutant SOD1 in NSC-34 cells

**Fig. 4:**
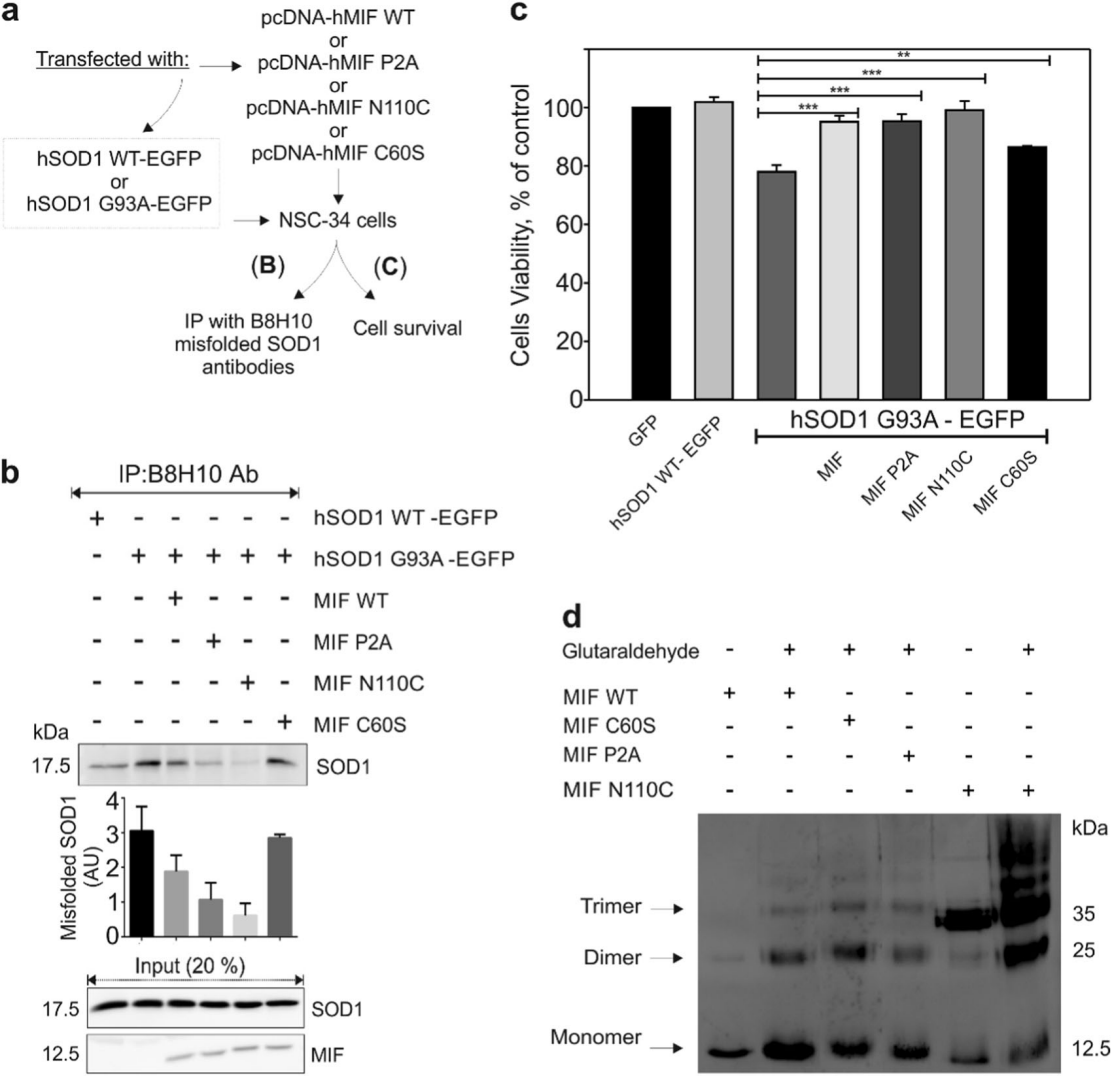
The catalytic activities of MIF and its oligomeric  transition are not decisive for its chaperone activity and its protective effect against mutant SOD1 toxicity in NSC-34 cells **a** A schematic representation of the experimental protocol. MIF-dependent inhibition of misfolded SOD1 accumulation and toxicity was tested in motor neuron-like NSC-34 cells, which were transfected to express the human wild-type SOD1 (SOD1^WT^–GFP) or the human mutant SOD1^G93A^–GFP with wild-type MIF (MIF^WT^), MIF^C60S^, MIF^P2A^, or MIF^N110C^. The cells were then subjected to an immunoprecipitation assay (**b**) or to a cell-survival assay (**c**). **b** Misfolded SOD1 was detected by immunoblotting of immunoprecipitates produced with the B8H10 antibody, which recognizes misfolded SOD1. EGFP-tagged wild-type or mutant human SOD1 levels were determined by immunoblotting in the initial cytosolic fractions. The presence and absence of MIF are represented by plus and minus signs, respectively. This immunoblot is representative of three different independent experiments. **c** A cell-survival analysis was performed with the CellTiter 96 AQ_ueous_ one-solution cell proliferation assay with ELISA at 490 nm. Quantitative analysis from triplicates of different biological experiments was performed with a Student’s *t*-test (*n* = 3); **p < 0.01, ***p < 0.001. **d** SDS-PAGE and immunoblot of recombinant MIF^WT^, MIF^C60S^, MIF^P2A^, and MIF^N110C^ after crosslinking with glutaraldehyde. Recombinant MIF^WT^ and mutant proteins at a concentration of 10 µM were crosslinked with 1% glutaraldehyde and samples electrophoresed as indicated. Wild-type MIF and non-crosslinked samples were analyzed for comparison. The monomer, dimer and trimer oligomers are indicated. +, sample crosslinked prior to electrophoresis; -, non-crosslinked control. This is one representative experiment out of three independent experiments performed.

MIF exhibits evolutionarily conserved catalytic tautomerase^29^ and thiol-oxidoreductase^30^ activities, but the physiologic substrates of these activities have remained elusive. To determine whether the ability of MIF to suppress the accumulation of misfolded SOD1 and protect against mutant SOD1 toxicity depends on these activities, we co-transfected NSC-34 cells with a human wild-type (SOD1^WT^) or mutant (SOD1^G93A^) SOD1 transgene together with either wild-type or point-mutated variants of MIF that completely lack tautomerase (MIF^P2A^) or oxidoreductase (MIF^C60S^) activities^29,30^. In addition, we transfected cells with a cysteine mutant of MIF (MIF^N110C^), which covalently locks MIF into a trimeric conformation by forming a disulfide bridge with Cys-81 of an adjacent subunit^31^, thus preventing equilibria between MIF trimers, dimers, and monomers^31–33^ (Fig. 4a).

In the absence of transfected MIF, misfolded SOD1 accumulated and could be detected by immunoprecipitation with the B8H10 antibody, which recognizes a wide range of misfolded SOD1 mutants, including SOD1^G93A^ (Fig. 4b). By contrast, expressing SOD1^G93A^ simultaneously with MIF^WT^ or with any of the three MIF mutants reduced (although to a lesser extent in the case of MIF^C60S^) the levels of misfolded SOD1 (Fig. 4b), but did not affect the overall level of SOD1 expression. In addition, while expressing SOD1^WT^ did not affect cell survival, expressing mutant SOD1^G93A^ reduced cell survival by approximately 25%. This toxic effect was reversed by co-expressing MIF^WT^ or any of the three MIF mutants (again with a smaller effect in MIF^C60S^) in these cells (Fig. 4c), suggesting that the tautomerase activity of MIF, its thiol-oxidoreductase activity, or the respective residues, as well as its oligomeric transition states are not required for its chaperone activity and cytoprotective effects. To further support this notion, we performed crosslinking experiments using glutaraldehyde as described previously^32^. Crosslinked MIF^WT^, MIF^P2A^ and MIF^C60S^ were found as a mixture of monomeric, dimeric, and trimeric forms, whereas mostly monomeric MIF^WT^ was detected when MIF was electrophoresed without prior crosslinking (Fig. 4d). In contrast to MIF^WT^, MIF^P2A^ and MIF^C60S^, the mutant MIF^N110C^ was found mostly as a trimeric form when electrophoresed without prior crosslinking, as expected^31^ (Fig. 4d).

### The locked-trimeric mutant MIF^N110C^ strongly suppresses the formation of mutant SOD1 amyloid fibrils

**Fig. 5:**
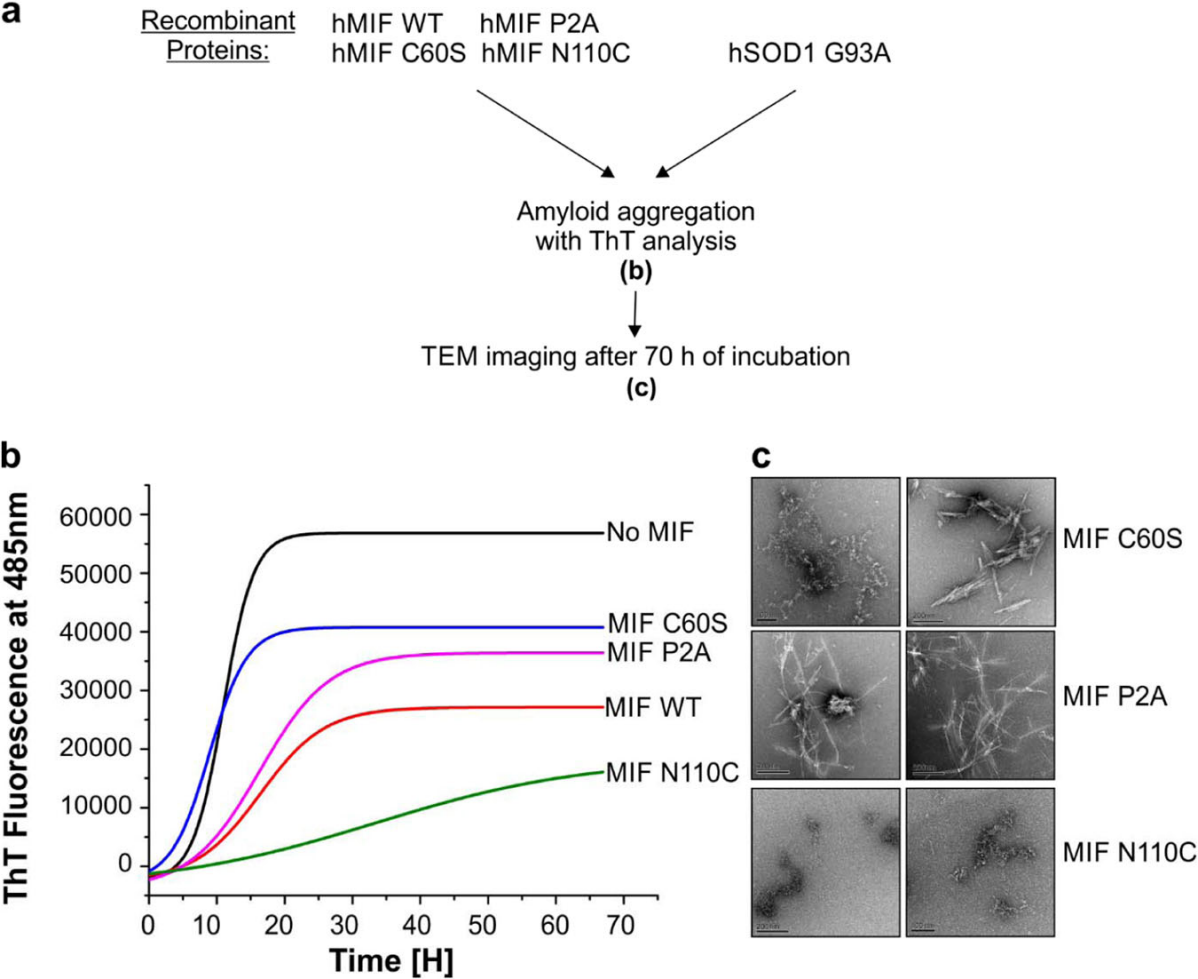
The locked-trimeric mutant MIF^N110C^ completely suppresses amyloid fibril formation of the mutant SOD1 **a** Experimental protocol used for determining whether purified recombinant MIF^WT^ or MIF mutants suppress the amyloid aggregation of misfolded SOD1, as detected by ThT and TEM . **b** ThT fluorescence was monitored during the incubation (37 °C with continuous shaking) of mutant SOD1^G93A^(50 µM), either without (black) or with recombinant MIF^WT^ (red), MIF^C60S^ (blue), MIF^P2A^(pink), or MIF^N110C^(green), all at a 10 µM concentration. Fluorescence was normalized to the maximal ThT fluorescence intensity that was elicited by SOD1^G93A^ alone. Data indicate the average of 30–50 replicates performed from three independent experiments. Fluorescence was fitted to Boltzmann sigmoidal equation using OriginPro 8.5 software. **c** TEM images of a SOD1^G93A^ (50 µM) solution after a 68-h incubation at 37 °C with continuous shaking. SOD1^G93A^ was incubated in the presence of recombinant MIF^C60S^, MIF^P2A^, or MIF^N110C^ at a molar ratio of 5:1.

**Fig. 6:**
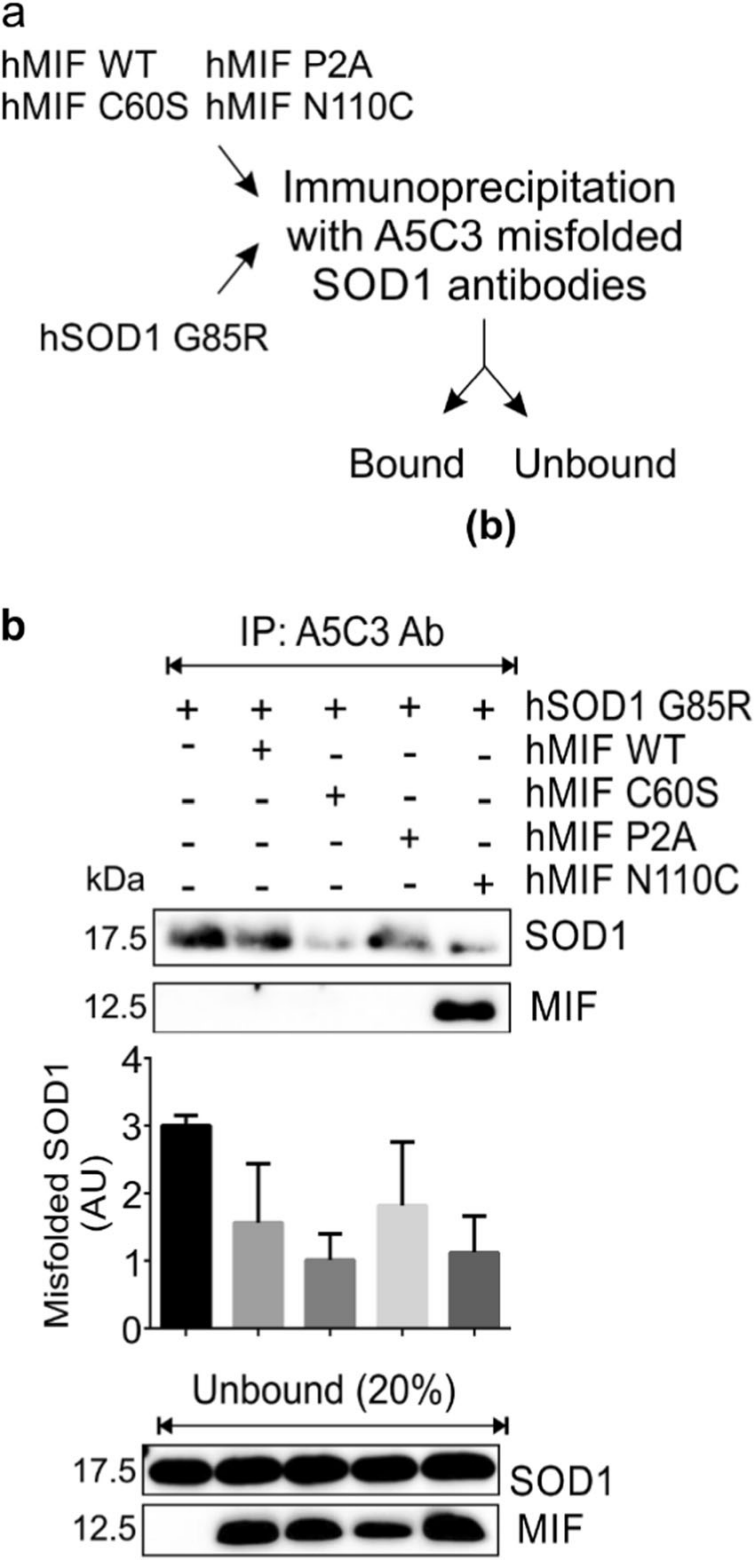
MIF inhibition of mutant SOD1 misfolding is independent of its enzymatic activities or its normal oligomeric transitions **a** Schematic diagram of the experiment. **b** The accumulation of misfolded SOD1 was determined by immunoblotting of immunoprecipitates with the A5C3 antibody after incubating recombinant hSOD1^G85R^ (4 µg) with (+) or without (–) recombinant MIF^WT^, MIF^C60S^, MIF^P2A^, or MIF^N110C^ (all at 2 µg). Immunoblotting was used to determine the levels of SOD1 and MIF that remained in the unbound fraction of each immunoprecipitation assay. This immunoblot is representative of three independent experiments.

We next tested whether the formation of mutant SOD1 amyloid fibrils can be prevented by co-incubation with MIF^WT^, MIF^C60S^, MIF^P2A^, or MIF^N110C^, all of which had a protective effect in the NSC-34 cells. To this end, we incubated the human SOD1^G93A^ apo-protein in the absence or presence of recombinant MIF^WT^, or its mutants (Fig. 4d) and measured ThT fluorescence over time. In order to confirm that all the proteins that we used were soluble at the concentration used, we performed a turbidity assay to measure protein aggregate formation. At 50 µM of recombinant MIF, we observed that MIF^WT^, MIF^C60S^, MIF^P2A^, and MIF^N110C^ formed insoluble aggregates to some extent when incubated at 37 ˚C (about 80, 50, 75, and 60% of the protein remained in the soluble fraction, respectively) (Fig. S3a and b), therefore we reduced the working concentration of MIF to 10 µM. At this concentration, no insoluble aggregates were formed (Fig. S3c, Fig. 2d). In the absence of MIF, the typical exponential rise in ThT fluorescence was observed after the typical lag time. This increase was strongly suppressed when the SOD1^G93A^ mutant was incubated in the presence of recombinant MIF^WT^, MIF^C60S^, or MIF^P2A^ (Fig. 5a, b). Of note, the locked-trimeric mutant MIF^N110C^ prevented the rise in ThT fluorescence, suggesting a strong inhibition in the formation of SOD1 amyloid aggregates (Fig. 5b). This finding was validated by TEM images (Fig. 5c). Similar to the addition of MIF^WT^, addition of MIF^N110C^ completely changed the pattern of SOD1 aggregates to amorphous aggregates. In contrast, after addition of MIF^C60S^ or MIF^P2A^, a mix of amyloid fibrils and amorphous aggregates could be visualized (Fig. 5c). Furthermore, misfolded SOD1 immunoprecipitation using an A5C3 antibody was inhibited in the presence of recombinant MIF^WT^ or of any of the three MIF mutants (Fig. 6). Surprisingly, in contrast to MIF^WT^, which does not co-precipitate with misfolded SOD1, the locked trimeric mutant MIF^N110C^ strongly co-precipitated with misfolded SOD1 (Fig. 6), suggesting a higher affinity of SOD1 to the locked trimeric form of MIF.

### MIF^N110C^ directly interacts with apo-SOD1 with higher affinity than MIF^WT^ as determined by real time surface plasmon resonance (SPR)

**Fig. 7:**
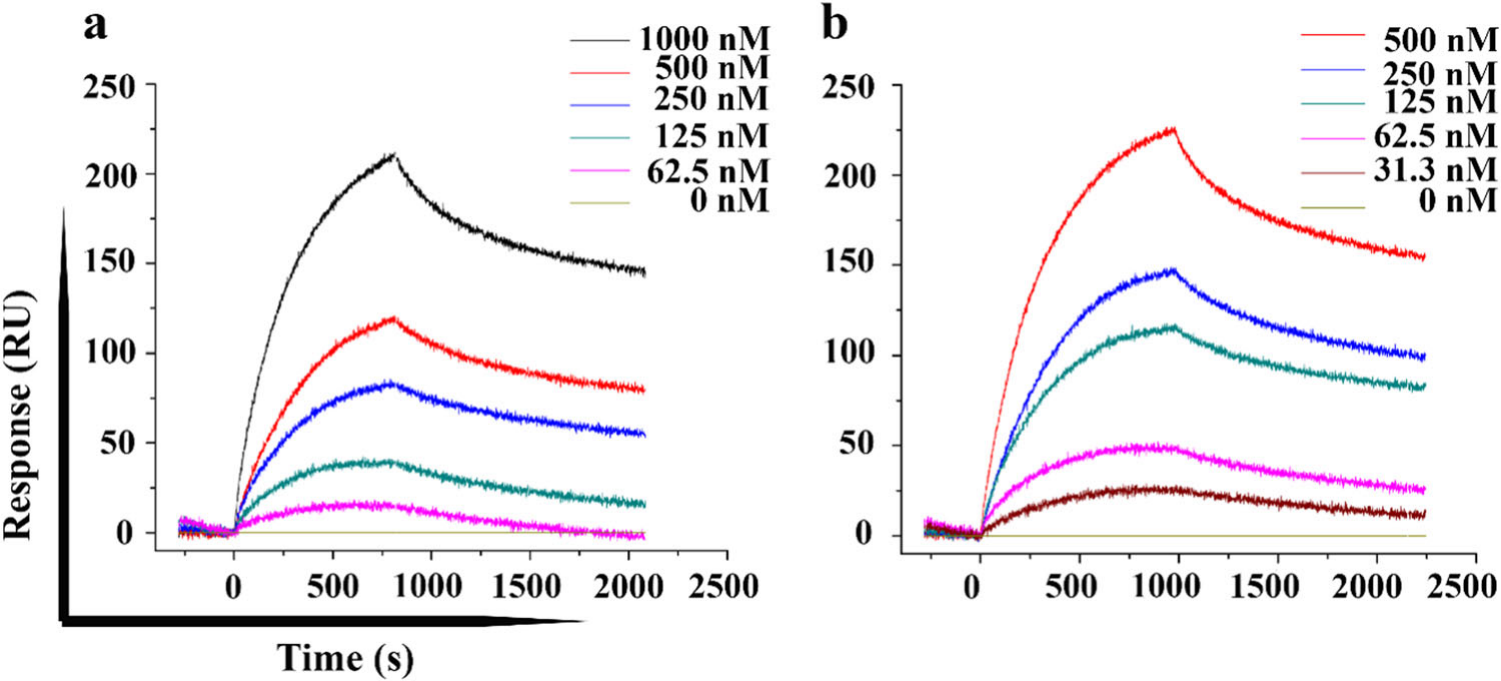
The locked trimer MIF^N110C^ binds apo-SOD1 protein with higher affinity than wild type MIF SPR binding sensograms for the interactions of Apo SOD1^WT^ with (**a**) MIF^WT^ and (**b**) mutant MIF^N110C^. Purified Apo SOD1^WT^ proteins were used as the ligand and recombinant MIF^WT^ and MIF^N110C^ were used as analytes. Concentrations of analytes are mentioned in the image. The sensograms shown are representative of five different experimental sets. The values are statistically valid since chi-square is less than the 10% of Rmax in the fitted graphs.

Recombinant MIF^WT^ and MIF^N110C^ were purified and their interactions with recombinant SOD1 demonstrated using SPR technology (Fig. 7). Poly-histidine tagged apo-SOD1 or HTB1 (as negative control) were immobilized on the surface of the SPR biosensor chip. Increasing concentrations (31–1000 nM) of MIF^WT^ or MIF^N110C^ were injected onto the sensor chips and binding to apo-SOD1 or HTB1 was monitored. Both MIF^WT^ and MIF^N110C^ strongly bound to immobilized apo-SOD1 in a concentration- and time-dependent manner (Fig. 7a, b). However, MIF^N110C^ showed a slower dissociation from the immobilized apo-SOD1 protein. The apparent binding affinities of MIF^WT^ and MIF^N110C^ to apo-SOD1 were derived from the sensogram of the steady-state values and calculated to be 1.3 ± 0.27 µM, and 0.548 ± 0.18 µM for MIF^WT^ and MIF^N110C^, respectively. The results thus demonstrate direct and specific interaction of MIF^WT^ and MIF^N110C^ with apo-SOD1, with a higher affinity for MIF^N110C^.

## Discussion

One of the most critical standing questions in the field of neurodegenerative diseases is the enigma of what determines the selective death of specific neuronal populations. In ALS cases related to mutant SOD1, the second most common form of familial ALS, the degeneration of motor neurons is accompanied by the specific misfolding of mutant SOD1. Several groups have reported the accumulation of misfolded SOD1 also in sporadic cases of ALS^10,34–40^, although other groups came to the opposite conclusion^41–46^. Misfolded SOD1 associates with mitochondria and ER membranes, and aggregates only in affected tissues. Recently, we demonstrated that the association of mutant SOD1 with intracellular membranes can be inhibited by the cytosolic multi-functional mediator MIF^47^, which significantly suppresses the accumulation of misfolded SOD1^24^. Moreover, we showed that MIF levels are low within motor neurons, as compared with other cell types, and that increasing MIF levels in mutant SOD1 expressing motor neurons can extend their survival^24^. Finally, we showed, in vivo, that reducing the chaperone activity of MIF plays a pivotal role in the accumulation of misfolded SOD1 and in its consequential toxicity^25^. By breeding MIF-null mice with mutant SOD1^G85R^ mice, we showed that mutant SOD1 mice that lack MIF expression have an accelerated disease onset and progression, and a shorter survival time than their mutant SOD1 littermates. This effect was accompanied by an increase in the accumulation of misfolded SOD1 and its association with mitochondria and ER membranes, apparent even in early stages and before the appearance of symptoms^25^.

In the current study, we deal with one of the most important unresolved questions; how does MIF protect motor neurons from toxic misfolded SOD1 accumulation? Specifically, we demonstrate that MIF suppresses the toxicity of misfolded SOD1 in motor neuron-like cells by directly interacting with misfolded SOD1 and by changing the aggregation pattern from amyloid aggregates to amorphous ones. In addition, we show that MIF inhibits the export of mutant SOD1 from the nucleus to the cytoplasm in these neuronal cells.

MIF was one of the first cytokines to be described^48^. Intracellularly, MIF was previously shown to act as a chaperone protein^49^ and as a thiol-protein oxidoreductase^30^, in addition to possessing a tautomerase activity–which is observed only in vitro and whose physiological relevance is unclear^29^. Here we show that these catalytic activities are not required for the protective chaperone effect of MIF.

Although the crystal structure of MIF indicates that it is a homo-trimer, MIF monomers and dimers have also been identified^32,33,50–53^. To determine which oligomeric form is necessary for its chaperone activity, we used MIF^N110C^—a previously reported cysteine mutant of MIF, which covalently locks MIF into its trimeric state^31,33^. Notably, while MIF^N110C^ retains partial catalytic activity and receptor binding to CD74, it loses its CD74-dependent cellular signaling ability as a cytokine^31^. Surprisingly, in our study, MIF^N110C^, which exists mostly in a trimeric form as was previously shown^33^, in contrast to MIF^WT^, not only retained its protective function, but it also showed a strong inhibition of SOD1 amyloid aggregate formation which was more pronounced than that of MIF^WT^. Furthermore, while MIF^WT^, which consists of mixtures of trimeric, dimeric and monomeric species, does not coprecipitate with misfolded SOD1 and shows lower affinity for apo-SOD1 in SPR analysis, the locked-trimeric mutant has a higher affinity for misfolded SOD1, as can be observed by SPR analysis or by immunoprecipitation using specific conformation antibodies for misfolded SOD1. This can be explained by the increased stability of the MIF trimer compared to the monomer species^33^. Thus, this mutant via its stable conformation, may have a beneficial effect intracellularly such as in the case of misfolded SOD1 binding. On the other hand, Fan and colleagues showed that the locked-trimeric mutant functions as an antagonist for MIF by binding to the MIF receptor CD74 without activating it, probably due to the absence of full flexibility of the locked trimeric mutant^31^. Altogether, these findings suggest that the trimeric form is the preferred one for the MIF chaperone-like activity. Moreover, MIF chaperone activity can be dissociated for the first time from its cellular signaling role as a pro-inflammatory cytokine.

It is well established that wild-type and mutant SOD1 may aggregate into amyloid fibrils under certain conditions^54–60^. Several studies have demonstrated the general toxicity of amyloid aggregates^61–64^. We show that MIF decreased the amyloid aggregation of SOD1^G93A^ and SOD1^G85R^ in a dose-dependent manner, and that its presence changed the aggregation pattern from fibril-like aggregates to amorphous disordered aggregates. This MIF-mediated change in the aggregation pathway of misfolded SOD1 is an important piece of the puzzle, since it was shown that the fibril-like amyloid aggregates of SOD1 are more toxic to the cells than non-fibril, disordered aggregates^54^. The change in aggregation pathway may thus explain the ability of MIF to rescue neuronal cells expressing mutant SOD1. Furthermore, the glycine–alanine dipeptide repeats from C9orf72 hexanucleotide expansions, which underlie the most common form of familial ALS, were shown to form toxic amyloid aggregates with a cell-to-cell transmission property^61,64,65^. In support of our current findings, it was shown that mutating the poly(GA) protein changes its aggregation pathway from fibrils to amorphous aggregates, which also suppressed its toxicity^64^.

Recently, Woerner and colleagues have used artificial β-sheet proteins to show that their cytosolic aggregates affect the nucleocytoplasmic transport of other proteins and RNA. However, the same β-sheet proteins had no effect when forming aggregates in the nucleus^63^. Based on this evidence that aggregates in the nucleus are less perturbing than their cytosolic counterparts, our results showing that MIF inhibits the sequestration of mutant SOD1 from the nucleus to the cytosol may provide an additional explanation for MIF’s protective effect especially considering the fact that nucleocytoplasmic transport defects play a central role in ALS pathogenesis^64,66–70^. Additionally, a study by Zhong et al., proposed that the nuclear clearance of misfolded SOD1 is a protective mechanism of the cell to fight against misfolded SOD1 toxicity in the nucleus^27^. They propose that the misfolding of SOD1 exposes an NES-like sequence, which is normally buried in the correctly folded wild type SOD1, allowing the sequestration of misfolded SOD1 to the cytoplasm^27^. We suggest that MIF’s inhibition of the nuclear export of misfolded SOD1 is due to its chaperone activity in the nucleus, preventing the exposure of the NES-like sequence. In this regard, it was recently shown that MIF is recruited to the nucleus by apoptosis-inducing factor (AIF), where it can also function as a nuclease^71^. Thus, whether the alleviation of misfolded SOD1 toxicity results from reducing its accumulation in the nucleus, by inhibiting its export to the cytosol, by modification of its toxic properties (such as aggregation), or by a combination of all-together still needs to be clarified.

In addition, with the recently proposed mechanism for cell-to-cell spreading of misfolded SOD1 as a means of disease propagation^72–74^, it will be important to determine, in future studies, whether MIF may act to limit such spreading considering the potential role of MIF as a chaperone and its extracellular localization under certain conditions.

**Fig. 8:**
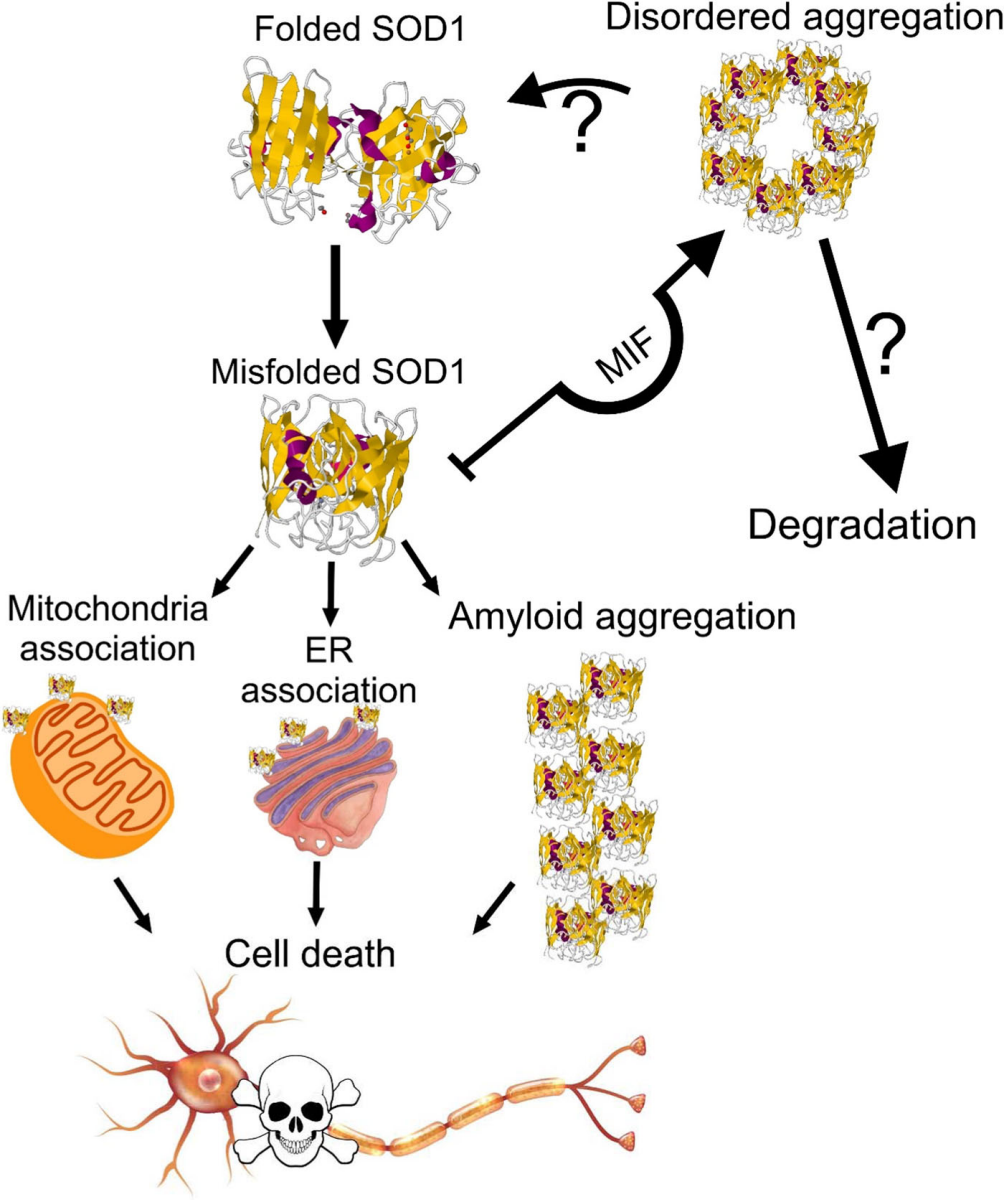
Proposed model of the chaperone-like activity of MIF A fraction of mutant SOD1 or wild type SOD1 under stress conditions accumulates as a toxic misfolded protein. This misfolded protein associates with mitochondrial or ER membranes, thereby causing mitochondrial dysfunction or ER stress, respectively, leading to cell death. Alternatively, the misfolded SOD1 proteins may aggregate as toxic amyloid fibrils that cause cell death. By directly interacting with the misfolded SOD1, MIF prevents it from accumulating and associating with intracellular membranes, thereby reducing its toxic effect. In addition, MIF inhibits SOD1 amyloid fibril formation and promotes instead, the formation of less toxic amorphous aggregates.

In conclusion, we propose a novel mechanism for MIF’s protective chaperone activity (as described in Fig. 8), independent of its catalytic and signaling functions. The elucidation of the exact mechanism by which MIF suppresses the accumulation of misfolded SOD1, inhibits its association with intracellular membranes, suppresses amyloid aggregation and rescues cells from mutant SOD1 toxicity, requires further investigation. This alternative mechanism proposed in our current research together with an in depth understanding of its essence has the potential to pave the way for the development of therapies for ALS based on MIF and its derivatives.

## Materials and methods

### Cell culture and plasmids

To generate the pcDNA-hMIF, pcDNA-hMIF^C60S^, pcDNA-hMIF^P2A^, and pcDNA-hMIF^N110C^ vectors, the cDNA of human MIF was amplified by polymerase chain reaction (PCR) and inserted into the pcDNA3.1(-) plasmid using the BamHI and XbaI sites. pCI-hSOD1^WT^, pCI-hSOD1^G93A^, and pCI-hSOD1^G85R^ were generated by inserting human SOD1 constructs into the pCI-NEO vector (Promega), between the EcoRI and the NotI sites. hSOD1^WT^–EGFP and mutant hSOD1^G93A^–EGFP were obtained from Jean-Pierre Julien (Laval University, Canada).

NSC-34 cells were grown at 37 °C and 5% CO_2_ in Dulbecco’s modified Eagle medium (DMEM) supplemented with 10% tetracycline-free fetal bovine serum (FBS), L-Glutamine (2 mM), and penicillin (100 U/ml)/streptomycin (0.1 mg/mL) (all reagents from Biological Industries). Transfection was performed by using TurboFect (Thermo) according to the manufacturer’s protocol. When co-transfections were performed, empty plasmids were transfected as controls. Cell viability was analyzed by using the CellTiter 96 AQ_ueous_ one-solution cell proliferation assay (Promega) and ELISA at 490 nm, according to the manufacturer’s protocol.

### Immunoprecipitation

Whole-cell extracts (100 µg) or purified proteins (4 µg) were solubilized in immunoprecipitation (IP) buffer [50 mM Tris-HCl (pH 7.4), 150 mM NaCl, 1 mM ethylenediaminetetraacetic acid (EDTA), 0.5% Nonidet P-40 plus 1X protease inhibitors] and incubated overnight with B8H10 or A5C3 (MediMabs) antibodies previously crosslinked to Dynabeads protein G (Invitrogen) with dimethyl pimelimidate (Pierce) according to the manufacturer’s instructions. The beads were magnetically isolated and washed three times with IP buffer. Samples were eluted by boiling in a 2 × sample buffer.

### Immunoblotting

Proteins were separated on 13% SDS-PAGE gel, transferred to nitrocellulose membranes, and probed with various antibodies, including goat anti-SOD1 (C-17; SCB), sheep anti-SOD1 (Calbiochem), monoclonal anti-VDAC/porin 31HL (Calbiochem), goat anti-MIF (N-18, SCB), rabbit anti-MIF (FL-115, SCB), rabbit anti-human SOD1 (ab52950, Abcam), and rabbit anti-VDAC (ab154856, Abcam). Horseradish peroxidase-conjugated anti-mouse, anti-rabbit, anti-sheep, or anti-goat IgG secondary antibodies (Jackson Immunochemicals) were used and detected by ECL (GE Biosciences).

## Protein expression and purification

### MIF purification

The pET-IIb plasmid containing human MIF cDNA was used to transform the *E. coli* BL21 (DE3) expression strain (Real Biotech). Three liters of culture were grown at 37 °C until the turbidity at 600 nm reached 0.4–0.6 OD. Isopropyl 1-thio-P-D-galactopyranoside (IPTG) was added to obtain a final concentration of 0.5 mM and the incubation continued at 37 °C overnight. Thereafter, bacteria were harvested by centrifugation (at 4700x*g* for 1.5 h) and the cell pellet (around 4.5 g for 600 mL) was resuspended to the ratio of 1 g:10 ml of MIF lysis buffer (20 mM Tris, 20 mM NaCl, 0.1 mM PMSF, and PI-complete cocktail tablets, pH 7.4). The bacteria were lysed by sonication (duration, 6 min; amplitude, 85%; on: 20 s; off: 40 s). Bacterial extract was centrifuged at 7000 RCF for 1.5 h. The supernatant was purified through a 0.45 µm filter, followed by a 0.22 µm filter (syringe-driven filter unit, Millex) and then subjected to Capto Q 5 mL anion exchange chromatography using the AKTA pure chromatography system (GE Healthcare). The Capto Q column was equilibrated with the MIF loading buffer (20 mM NaCl, 20 mM Tris, pH 7.4, 5 column volumes). The supernatant was loaded onto the column with a flow speed of 1 mL/min. MIF does not bind to the column under these conditions, and the flow-through fractions containing MIF (Peak UV omission on graph) were collected, combined and left overnight at 4 °C.

The following morning, the solution was centrifuged at 7000 RCF for 1 h, and the supernatant was examined using Capto S 5 mL cation exchange chromatography that had previously been equilibrated with the loading buffer (5 CV). The supernatant was loaded onto the column in gradual volumes of 7 ml at a flow speed of 0.5 ml/min. The Capto S column was then washed with the original MIF loading buffer causing weak elution. After the washing, MIF detached from the column as flow through. This loading and eluting procedure of MIF on the Capto S column was repeatedly done in small volumes. The peak fractions were loaded onto a SDS-PAGE gel (13%). The most purified MIF-containing fractions were combined; centrifuged at 4000 RCF for 20 min in centrifugal filter units 10 K (Merck-Millipore), quantified by Bradford assay, aliquoted and stored at −20 °C.

### Purification and evaluation of recombinant SOD1^WT^, SOD1^G93A^, and SOD1^G85R^ proteins

Sequences of human SOD1^WT^, SOD1^G93A^, and SOD1^G85R^ were optimized for codon usage in *E. coli*, cloned into pHIS1 vector^75^ and expressed as 6His-tagged (*N*-term) soluble proteins in BL21 cells. Cells were grown in LB medium containing 100 μg/mL ampicillin at 30 °C for 4 h and the expression of the recombinant SOD1 proteins was induced by the addition of 0.5 mM IPTG, followed by overnight incubation at 20 °C. Afterwards, bacteria were harvested by centrifugation (at 4700×*g* for 1.5 h) and the cell pellet (around 8 g for 600 mL) was resuspended in sonication buffer (50 mM Tris-HCl pH 7.5, 0.5 M NaCl, 2 mM β-mercaptoethanol, 10 mM imidazole, 1 mg per 1 ml lysozyme, 0.1 mM PMSF and PI- cOmplete cocktail tablets). After 30 min incubation on ice, the cells were disrupted by sonication (duration, 6 min; amplitude, 85%; on: 20 s; off, 40 s) in a loading buffer (50 mM Na^+^/phosphate, pH 7.6, 0.5 M NaCl, 2 mM β-mercaptoethanol, 10 mM imidazole) containing 1 mg/mL lysozyme and a protease inhibitor cocktail (Sigma, Israel).

To remove DNA, the crude extract was incubated on ice for 30 min in the presence of 100 U/mL bovine pancreas DNaseI (Sigma, Israel) and 5 mM MgSO_4_, followed by 1.5 h centrifugation (7000×*g*) at 4 °C. The supernatant was purified through a 45 µm filter, followed by a 22 µm filter (syringe-driven filter unit, Millex) and then subjected to a 5 mL HisTrap FF column (GE Healthcare Life Sciences, Sweden) equilibrated with the loading buffer. The column was washed (5 CV) with a washing buffer (50 mM Na^+^/phosphate, pH 7.6, 0.5 M NaCl, 2 mM β-mercaptoethanol, 20 mM imidazole) and the protein was eluted by a linear 20–400 mM imidazole gradient (10 CV). The peak fractions were dialyzed overnight at 4 °C against a storage buffer (50 mM Na^+^/phosphate, pH 7.6, 0.1 M NaCl, and 10% glycerol), concentrated by ultrafiltration (10 kDa cutoff, Millipore, USA), centrifuged at 110,000×*g* at 4 °C for 1 h using ultracentrifuge (Sorvall M120, Discovery), and stored at −20 °C until use. Protein concentration was measured by the Bradford method using bovine serum albumin as the standard.

### ThT aggregation assay for mutant SOD1

Prior to the aggregation assay, all mixtures containing protein were centrifuged at 10,000×*g* at 20 °C for 10 min using ultracentrifuge (Sorvall M120, Discovery). SOD1^G93A^ or SOD1^G85R^ (50 μM) with or without MIF (6.25–50 µM) were incubated in 200 μL of 10 mM Na^+^/phosphate buffer, pH 7.4, 150 mM NaCl, 5 mM EDTA and 10 mM TCEP in the presence of 20 μM Thioflavin T (Sigma Aldrich, Israel) in a black 96-well plate at 37 °C with fast continuous shaking using a SpectraMax Paradigm (Molecular Devices) ELISA reader. The fluorescence (Ex. 440 nm; Em. 485 nm) was measured at 10 min intervals.

### Analysis of SOD1 fibril formation by transmission electron microscopy (TEM)

Samples for TEM imaging were prepared as described elsewhere^76^. Briefly, at the end of the aggregation assay, 2.5 μL samples (diluted 5-fold) were deposited on a carbon-coated copper 300 mesh. After 1 min, the excess liquid was carefully blotted onto filter paper, which was then dried at ambient temperature for 1 min. Uranyl acetate (5 μL, 2%) was added to the grid and, after 1 min, the excess of the salt solution was carefully removed with a filter paper. The imaging was performed using a Tecnai G2 12 BioTWIN (FEI) transmission electron microscope with an acceleration voltage of 120 kV. Different magnifications were used, depending on the size of the aggregate. The visible features were sensitive to the electron beam exposure, indicating their organic origin.

### Surface plasmon resonance

The binding constants of MIF^WT^ and MIF^N110C^ mutant with apo-SOD1^WT^ protein, were determined by SPR spectroscopy on a ProteOn XPR36 (Bio-Rad, CA, USA). Poly-histidine tagged apo-SOD1^WT^ was immobilized on the surface of the tris-NTA HTG ProteOn chip. 1 µg of apo-SOD1^WT^ was immobilized on the chip in PBST buffer (PBS 300 mM NaCl, 0.05% Tween-20), to give 736 response units (RU). HTB1, Hyperthermophilic protein G B1 domain, (10 μg, 2068 RU) was immobilized on the chip as a negative control. Before each binding assay, the temperature was set at 25 °C. Soluble MIF wild type and MIF mutant (analytes) were then allowed to flow over the surface-bound apo-SOD1^WT^ separately at a flow rate of 25 µl/min. While the analyte was flowing over the surface for 13 min, the interactions of MIF^WT^ and MIF^N110C^ with apo-SOD1^WT^ was determined. The next step was to examine the dissociation of the protein, while allowing PBST to flow over the surface for 21 min at 25 µl/min. After each run, a regeneration step was performed with 50 mM NaOH at a flow rate of 100 μL/min. For each protein complex, a sensogram was generated from the RUs measured during the course of the protein–protein interaction subtracting the values of the HTB1 background channel. The dissociation constant (K_D_) was determined from the sensogram of the equilibrium-binding phase.

### Protein crosslinking with glutaraldehyde

Ten μM (micromolar) of recombinant wild-type or mutant MIF proteins were dissolved in 20 mM sodium phosphate buffer (pH 7.2) and incubated for 3 h in the presence of 1% glutaraldehyde^32^ at room temperature in constant rotation. The reaction was stopped by adding 25 mM NaBH_4_. Samples were then boiled in Laemmli sample buffer without β-mercapthoethanol and analyzed by 13% SDS-PAGE followed by immunoblot analysis with anti-MIF polyclonal antibody (FL-115).

### Statistics

Values are reported as mean ± SEM throughout. Student’s *t*-tests were used to compare two datasets, after confirming a normal distribution of the data by using the Shapiro-Wilk normality test. A one-way ANOVA with Tukey’s posthoc analysis was used to analyze the nucleus/cytoplasm SOD1 distribution. Significance was set at a confidence level of 0.05.

## Electronic supplementary material

Supplementary Figure 1
