# Supplementary material for: MIF inhibits the formation and toxicity of misfolded SOD1 amyloid aggregates: implications for familial ALS
Source: Cell Death Dis. 2018 Jan 25;9(2):107. doi: 10.1038/s41419-017-0130-4 (PMC5833700; doi:10.1038/s41419-017-0130-4)
Supplement: Supplementary file 1 — Supplementary Figure 1 [file 41419_2017_130_MOESM1_ESM.pdf]

Supporting Information

Supplementary figures

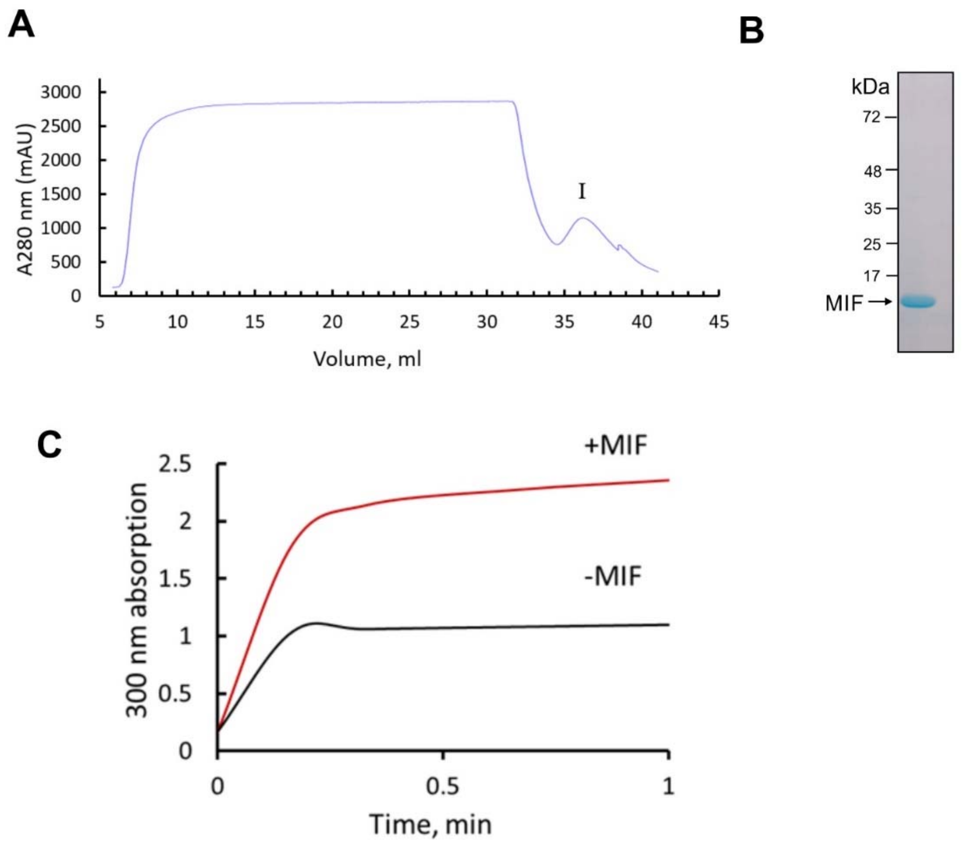

Fig. S1. Purification and activity of recombinant MIF. (**a**) Ion exchange chromatography using a Capto S column of supernatants containing recombinant MIF. The peak of interest, which represents MIF enrichment, is labeled as I. (**b**) SDS-PAGE of peak I from the ion exchange chromatography. (**c**) HPP tautomerization rate was observed as the increase in absorption at 300 nm with (red) or without (black) the addition of 100 nM recombinant MIF.

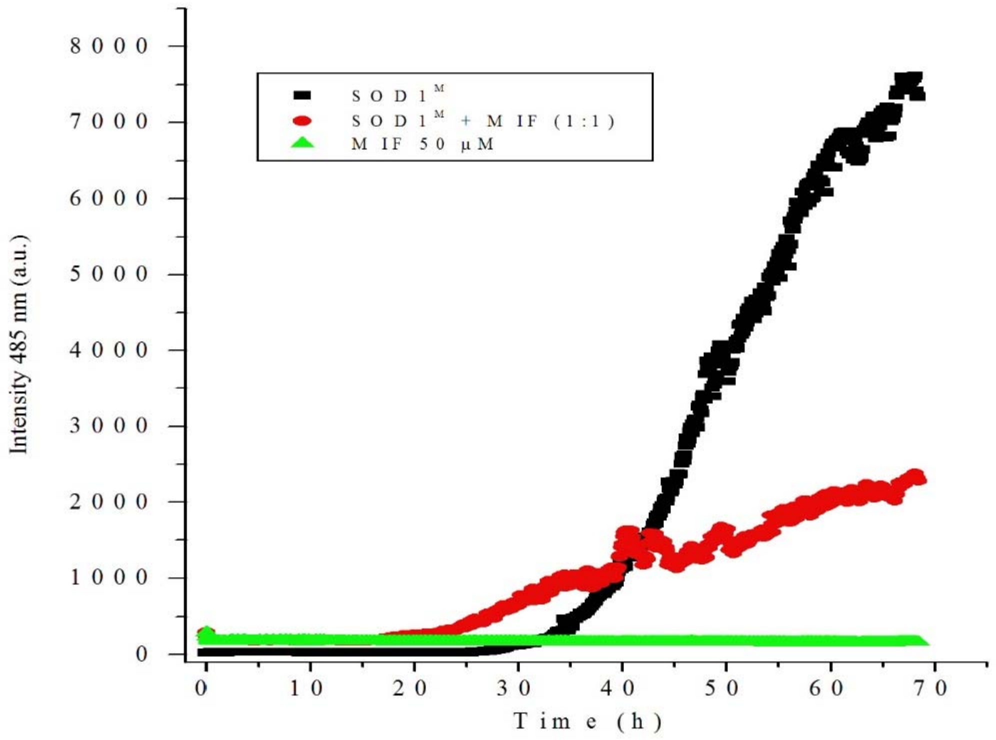

Fig. S2. Recombinant MIF does not form amyloid aggregates.
ThT fluorescence was monitored during the incubation of recombinant SOD1^{G93A} alone (black), recombinant MIF alone (green) or both of these proteins together (red) in 37 °C with continuous shaking.

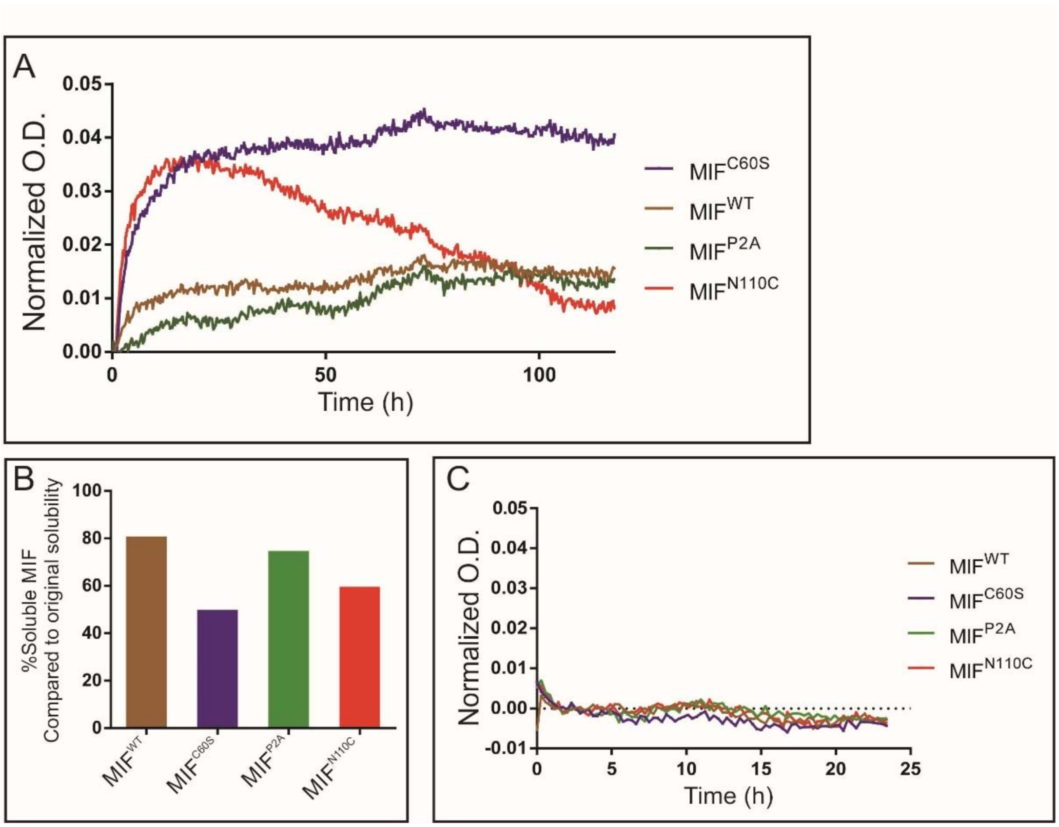

Fig. S3. MIF-dependent suppression of mutant SOD1 amyloid aggregation is not due to differences in solubility of the MIF variants. **(a)** Solubility of MIF^{WT} and MIF mutants (C60S, P2A, N110C) was tested at 50 μM, by observing absorption at 450nm for 5 days. MIF^{WT} and MIF^{P2A} show a gradual increase in absorption until reaching plateau at around 70 h. MIF^{C60S} shows rapid increase in absorption and reaching until reaching plateau at around 30 h. MIF^{N110C} absorption is similar to MIF^{C60S}, yet insoluble fraction aggregate causing a seemingly decrease in absorption (but not in solubility). **b**. Solubility percentage deduced from the MIF variants concentrations after 5 days of incubation. **c**. Solubility of MIF^{WT} and MIF mutants (C60S, P2A, N110C) was tested at 10 μM, a lower concentration reached by the most insoluble MIF variant at plateau. All MIF variants show no apparent increase in absorption, indicating no insoluble fractionation at this concentration.
